# Supplementary material for: Concurrent Drought and Temperature Stress in Rice—A Possible Result of the Predicted Climate Change: Effects on Yield Attributes, Eating Characteristics, and Health Promoting Compounds
Source: Int J Environ Res Public Health. 2019 Mar 22;16(6):1043. doi: 10.3390/ijerph16061043 (PMC6465994; doi:10.3390/ijerph16061043)
Supplement: Supplementary file 1 [file ijerph-16-01043-s001.pdf]

Supplementary Materials

Table S1. Means of grain yield plant^{-1} and grain yield components of rice cultivars grown at low (26/23 °C) or high temperature (27/30 °C) and subjected to different drought treatments.

Temperature	Drought	Cultivar	Panicles Plant^{-1}	Panicle Length (cm)	Spikelets Spike^{-1}	Spikelet Sterility (%)	Grain Yield Plant^{-1} (g)
Low	D0	Ingwizabukungu	0.6	16.3	6.0	51.5	1.9
Low	D0	Intsindagirabigega	3.2	19.9	8.3	19.2	13.3
Low	D0	Jyambere	0.0	0.0	0.0	NA	0.0
Low	D0	Mpembuke	6.4	25.5	9.2	36.1	6.2
Low	D0	Ndamirabahinzi	1.0	25.1	8.1	29.8	3.7
Low	D0	Nemeyubutaka	2.4	24.7	9.7	43.7	4.8
Low	D0	Zong geng	0.6	31.0	10.5	25.0	5.0
Low	DS	Ingwizabukungu	0.4	24.3	9.7	63.3	1.2
Low	DS	Intsindagirabigega	1.0	19.0	10.0	27.7	1.4
Low	DS	Jyambere	5.6	24.7	10.3	88.1	1.5
Low	DS	Mpembuke	0.0	0.0	0.0	NA	0.0
Low	DS	Ndamirabahinzi	0.0	0.0	0.0	NA	0.0
Low	DS	Nemeyubutaka	1.6	23.9	7.8	41.5	1.8
Low	DS	Zong geng	0.4	28.0	9.0	5.5	1.4
Low	DST	Ingwizabukungu	0.2	25.5	7.0	100.0	0.0
Low	DST	Intsindagirabigega	3.4	23.6	8.3	52.8	4.4
Low	DST	Jyambere	6.2	21.0	8.8	52.0	3.4
Low	DST	Mpembuke	1.2	26.3	9.7	100.0	0.0
Low	DST	Ndamirabahinzi	0.2	21.0	7.0	58.8	0.5
Low	DST	Nemeyubutaka	1.2	26.7	9.7	46.2	2.5
Low	DST	Zong geng	0.2	21.0	11.0	100.0	0.0
Low	DT	Ingwizabukungu	0.4	22.0	7.5	33.8	1.0
Low	DT	Intsindagirabigega	1.2	28.5	11.8	45.2	5.1
Low	DT	Jyambere	0.6	17.0	5.8	25.7	0.9
Low	DT	Mpembuke	0.6	22.0	7.3	95.9	0.5
Low	DT	Ndamirabahinzi	0.0	0.0	0.0	NA	0.0
Low	DT	Nemeyubutaka	1.4	23.0	9.2	46.6	2.4
Low	DT	Zong geng	0.0	0.0	0.0	0.0	0.0
Low	DTR	Ingwizabukungu	0.2	19.0	5.0	100.0	0.0
Low	DTR	Intsindagirabigega	2.2	19.8	8.2	63.8	3.0
Low	DTR	Jyambere	0.8	16.0	6.0	60.8	1.1
Low	DTR	Mpembuke	0.0	0.0	0.0	NA	0.0
Low	DTR	Ndamirabahinzi	0.6	23.7	10.3	98.6	0.2
Low	DTR	Nemeyubutaka	0.0	0.0	0.0	NA	0.0
Low	DTR	Zong geng	0.0	0.0	0.0	NA	0.0
Low	DR	Ingwizabukungu	1.2	18.1	7.5	51.5	1.0
Low	DR	Intsindagirabigega	2.2	19.6	7.6	51.5	1.6
Low	DR	Jyambere	0.0	0.0	0.0	NA	0.0
Low	DR	Mpembuke	0.0	0.0	0.0	NA	0.0
Low	DR	Ndamirabahinzi	0.6	22.3	6.3	83.3	0.5
Low	DR	Nemeyubutaka	0.0	0.0	0.0	NA	0.0
Low	DR	zong geng	0.0	0.0	0.0	NA	0.0
Low	DSTR	Ingwizabukungu	0.0	0.0	0.0	NA	0.0
Low	DSTR	Intsindagirabigega	2.0	23.3	7.7	73.7	2.7
Low	DSTR	Jyambere	2.0	24.4	7.8	58.8	0.9
Low	DSTR	Mpembuke	1.2	28.8	11.7	51.8	3.0
Low	DSTR	Ndamirabahinzi	0.2	25.0	7.0	63.8	0.6
Low	DSTR	Nemeyubutaka	1.6	25.3	8.3	56.0	2.7
Low	DSTR	Zong geng	0.0	0.0	0.0	NA	0.0
High	D0	Ingwizabukungu	2.0	16.6	5.6	17.4	4.8
High	D0	Intsindagirabigega	1.6	21.3	9.0	34.5	5.7
High	D0	Jyambere	4.6	21.6	9.9	39.0	9.6
High	D0	Mpembuke	2.0	25.4	7.6	29.1	4.5
High	D0	Ndamirabahinzi	2.8	20.3	8.2	33.5	5.0
High	D0	Nemeyubutaka	1.4	22.1	7.5	33.3	4.8
High	D0	Zong geng	0.6	23.8	10.0	31.9	1.3

High	DS	Ingwizabukungu	0.6	14.7	4.8	20.4	1.2
High	DS	Intsindagirabigega	0.4	20.5	6.5	27.2	2.8
High	DS	Jyambere	4.4	24.8	9.3	19.8	4.4
High	DS	Mpembuke	0.6	23.3	8.3	36.5	1.2
High	DS	Ndamirabahinzi	0.0	0.0	0.0	NA	0.0
High	DS	Nemeyubutaka	0.0	0.0	0.0	NA	0.0
High	DS	Zong geng	0.0	0.0	0.0	NA	0.0
High	DT	Ingwizabukungu	1.4	19.0	5.7	50.1	1.2
High	DT	Intsindagirabigega	0.6	24.7	9.0	45.6	0.9
High	DT	Jyambere	4.0	21.6	7.8	22.1	5.3
High	DT	Mpembuke	1.2	18.5	7.0	61.5	1.5
High	DT	Ndamirabahinzi	1.8	25.0	7.6	49.7	3.7
High	DT	Nemeyubutaka	0.8	18.5	7.8	23.3	3.0
High	DT	Zong geng	0.0	0.0	0.0	NA	0.0
High	DTR	Ingwizabukungu	0.6	17.3	6.3	31.0	2.1
High	DTR	Intsindagirabigega	0.4	22.5	7.0	27.1	0.9
High	DTR	Jyambere	0.4	8.5	4.5	59.1	0.9
High	DTR	Mpembuke	0.6	20.0	6.5	26.9	1.3
High	DTR	Ndamirabahinzi	0.4	22.0	7.5	41.5	1.3
High	DTR	Nemeyubutaka	0.4	9.5	8.5	45.1	1.4
High	DTR	Zong geng	0.0	0.0	0.0	NA	0.0
High	DR	Ingwizabukungu	1.6	15.8	7.2	27.5	4.7
High	DR	Intsindagirabigega	1.4	20.2	8.6	46.1	1.2
High	DR	Jyambere	0.4	16.0	8.0	26.4	1.3
High	DR	Mpembuke	0.6	24.0	9.0	40.3	0.5
High	DR	Ndamirabahinzi	1.0	20.0	7.7	49.3	0.5
High	DR	Nemeyubutaka	1.8	15.7	7.2	54.8	1.7
High	DR	Zong geng	0.0	0.0	0.0	NA	0.0

D0: plants were well-watered along the growing cycle; DS: drought stress at seedling stage; DST: drought stress at seedling and tillering stages, DT: drought at tillering stage, DTR: drought at tillering and reproductive stages, DSTR: repeated drought at every developmental stage.

NA: spikelet sterility could not be evaluated because the plants died before flowering stage

Table S2. Means of quality characteristics: amylose content (AmC), gel consistency (GC), gelatinisation temperature (GT), total protein content (Prot), total phenolic content (TPC) and total antioxidant capacity (TAC) of rice cultivars grown at low (26/23 °C) or high temperature (27/30 °C) and subjected to different drought treatments.

Temperature	Drought	Cultivar	AmC (%)	GC (mm)	GT (°C)	Prot (%)	TPC (GAE 100g^{-1} DW)	TAC (μmol Fe2+ g^{-1} DW)
Low	D0	Ingwizabukungu	17.3	94.5	79.4	14.4	204.6	11.1
Low	D0	Intsindagirabigega	14.5	30.5	76.8	15.5	165.9	6.1
Low	D0	Mpembuke	8.3	84.5	76.4	13.3	351.3	69.5
Low	D0	Nemeyubutaka	4.1	44.5	76.3	13.9	157.1	8.3
Low	D0	Zong geng	8.8	96.5	68.1	8.6	148.1	11.4
Low	DS	Ingwizabukungu	8.8	71.5	75.5	14.1	169.3	7.5
Low	DS	Jyambere	9.4	34.0	76.9	16.6	136.5	9.6
Low	DS	Nemeyubutaka	7.3	82.5	77.2	14.0	292.3	46.1
Low	DS	Zong geng	13.9	34.5	69.1	9.0	248.7	15.1
Low	DST	Intsindagirabigega	25.6	76.5	74.6	12.7	305.7	7.1
Low	DST	Jyambere	8.3	29.5	76.6	14.2	236.4	3.4
Low	DST	Nemeyubutaka	6.1	33.0	75.8	13.2	249.0	6.9
Low	DT	Intsindagirabigega	19.9	95.5	77.4	13.9	190.6	4.3
Low	DT	Nemeyubutaka	13.3	32.5	75.9	13.2	215.6	9.3
Low	DTR	Intsindagirabigega	17.6	98.0	78.4	11.5	187.2	5.6
Low	DR	Intsindagirabigega	12.1	98.5	77.0	15.8	211.1	4.5
Low	DSTR	Intsindagirabigega	10.3	86.0	75.2	13.2	91.0	5.3
Low	DSTR	Jyambere	5.0	30.5	76.2	12.9	66.8	4.3
Low	DSTR	Mpembuke	5.4	33.5	76.8	19.1	389.1	73.6
Low	DSTR	Nemeyubutaka	10.4	79.0	76.3	14.3	78.6	8.4
High	D0	Ingwizabukungu	5.0	88.0	80.1	17.4	84.7	3.5
High	D0	Intsindagirabigega	10.9	58.0	78.9	11.6	154.0	33.9
High	D0	Jyambere	9.3	31.5	81.8	14.4	72.7	3.4
High	D0	Mpembuke	5.4	75.5	79.7	12.1	276.1	59.1
High	D0	Ndamirabahinzi	6.3	32.0	79.4	12.7	294.6	52.7
High	D0	Nemeyubutaka	8.6	40.0	80.5	12.2	148.5	31.6
High	D0	Zong geng	12.8	34.5	73.9	6.7	120.6	15.3
High	DS	Ingwizabukungu	8.2	34.0	79.6	14.0	70.7	9.5
High	DS	Intsindagirabigega	18.0	97.5	80.0	9.9	67.7	4.9
High	DS	Jyambere	10.4	85.0	81.0	12.0	68.8	2.1
High	DS	Mpembuke	7.6	31.5	79.8	10.7	316.8	58.2
High	DT	Ingwizabukungu	7.8	78.0	78.2	11.1	56.0	2.9
High	DT	Intsindagirabigega	8.5	36.0	80.0	12.3	47.6	3.2
High	DT	Jyambere	6.3	33.0	80.7	13.3	90.7	4.4
High	DT	Mpembuke	7.5	31.5	79.5	11.2	231.4	47.0
High	DT	Ndamirabahinzi	7.2	95.0	78.5	10.5	183.7	40.4
High	DT	Nemeyubutaka	8.0	31.5	79.8	9.7	176.0	41.1
High	DTR	Ingwizabukungu	14.8	44.0	79.9	11.0	82.5	4.1
High	DTR	Mpembuke	5.0	57.0	79.0	11.4	323.1	56.3
High	DTR	Ndamirabahinzi	7.3	71.5	80.3	11.9	343.0	52.0
High	DTR	Nemeyubutaka	5.3	32.0	80.5	12.3	83.9	6.1
High	DR	Ingwizabukungu	5.8	98.0	73.2	13.3	78.7	4.1
High	DR	Intsindagirabigega	12.8	84.5	78.8	10.2	114.3	4.6
High	DR	Nemeyubutaka	7.0	43.0	81.3	12.5	79.4	5.9

D0: plants were well-watered along the growing cycle; DS: drought stress at seedling stage; DST: drought stress at seedling and tillering stages, DT: drought at tillering stage, DTR: drought at tillering and reproductive stages, DR: drought at reproductive stage, DSTR: repeated drought at every developmental stage.
